# Innovative Nanotechnological Approaches in Trauma and Orthopaedic Surgery: A Comprehensive Review

**DOI:** 10.7759/cureus.72838

**Published:** 2024-11-01

**Authors:** Gaurav Jha, Surya Malasani, Ahmed Barakat, Siri Chandana Sola, Kashish Gera, Garima Gupta

**Affiliations:** 1 Trauma and Orthopaedics, University Hospitals of Leicester NHS Trust, Leicester, GBR; 2 Trauma and Orthopaedics, Guy's and St Thomas' NHS Foundation Trust, London, GBR; 3 Geriatrics, University Hospitals of Leicester NHS Trust, Leicester, GBR; 4 Internal Medicine, University Hospitals of Leicester NHS Trust, Leicester, GBR; 5 Cardiology, University Hospitals of Leicester NHS Trust, Leicester, GBR

**Keywords:** bone regeneration, coated implants, nanomedicine, orthopaedic research, orthopaedic surgery, regenerative orthopaedics, targeted drug delivery

## Abstract

The application of nanotechnology to health has been one of the revolutionizing factors in the field of trauma and orthopaedic surgery over the last decade. Advances in nanomedicine, in comparison to conventional modes of treatment, have influenced immensely the approach towards trauma and orthopaedic surgery and provided some unique answers to some very complex problems like bone reconstruction, soft tissue repair, and prevention of infection. The current narrative review intends to underpin an extensive analysis of modern applications and recent advances in nanotechnology-driven therapies in orthopaedics. Having leveraged unique properties inherent in nanoparticles and nanoscale materials, novel interventions, such as nanostructured scaffolds, drug delivery systems, and bioactive coatings, have flourished into a variety of promising means to enhance osseointegration, accelerate the healing process, and reduce postoperative complications. This review at once acknowledges the huge potential of these technologies and some of the problems impeding their wide-range clinical application, including long-term safety, main regulatory hurdles, and scale-up issues. The following review aims to give orthopaedic surgeons, researchers, and biomedical engineers an overview of the present status and perspectives for the future regarding nanomedicine in trauma and orthopaedic surgery, pointing out the expectations of a much-improved outcome in patients and overall quality of life.

## Introduction and background

Nanomedicine represents a novel approach within the realm of trauma and orthopaedic surgery. Innovative solutions and answers are being provided for the challenges that conventionally have troubled traditional modes of treatment. The application of nanoscale materials allows for enhanced control over drug delivery, improved biomaterial integration, and targeted therapeutic interventions, offering significant potential for improving patient outcomes in orthopaedic care​ [1,2]. With the continuously increasing global burden of both musculoskeletal injuries and degenerative diseases, there is an emerging need for novel therapeutic solutions capable of dealing effectively with issues related to the regeneration of bone, control of infection, and repair of soft tissues [3].

Conventional surgical techniques of orthopaedics are generally based on classic approaches and materials that, despite having achieved success, rarely deal with complex injuries sustained with a great amount of bone loss. Currently available therapeutic options to manage bone loss, particularly those involving trauma and infection, have limitations related to their effectiveness. Nanotechnology, defined by substances engineered at the nano level (1-100 nm), opens up new prospects for the future to help overcome some of the challenges mentioned above [4]. In fact, the use of nanomaterials such as nanoparticles (NPs), nanostructured scaffolds, and bioactive coatings has been shown to emulate such bone and cartilage structures and support cellular responses important in the process of healing ​[5,6]. For instance, nanostructured scaffolds can support cell proliferation and differentiation by simulating the natural bone hierarchy and extracellular matrix (ECM), leading to improved outcomes in injured tissues and bone regeneration and repair​ [7,8].

Recent research has focused on the potential of nanomedicine for solving some of the most pervasive problems in orthopaedic trauma, such as infection treatment and the enhancement of bone healing processes. Drug delivery systems based on NPs have been more specific in delivering therapeutic agents to infection sites and thus reduce dependence on systemic antibiotics, decreasing adverse effects [9]. The introduction of nanotechnology into the field of orthopaedic implants showed a great outlook for improving osseointegration (reducing the risks of implant failure) and inducing bone regeneration as promising ways to improve long-term surgical outcomes [2,10].

Despite these promising advancements, several challenges are impeding the clinical implementation of nanotechnology in orthopaedics, including regulatory barriers, concerns about the long-term safety of nanomaterials, and a lack of standardized methodologies [6]. Accordingly, this review addresses these gaps by giving an overview of the state of the art regarding recent advances in nanomedicine in the interdisciplinary field of trauma and orthopaedics, its potential applications, as well as the challenges to be overcome for wider clinical adoption.

## Review

The integration of nanotechnology has availed a multi-dimensional approach towards betterment in patient care in trauma and orthopaedic surgery. Unique properties developed at the nanoscale have enabled impressive enhancements in the bioactivity, mechanical strength, and therapeutic potential of orthopaedic interventions. A critical analysis of the available research reveals that nanomaterials have demonstrated remarkable capabilities in tissue engineering mimicking the composition and structure of various tissues, including bone, cartilage, and blood vessels [11].

Additionally, nanomaterials exhibit special physicochemical properties, such as a high surface area-to-volume ratio, increased reactivity, and a strong strength-to-weight ratio, making them ideal for developing advanced orthopaedic implants [12]. These nanomaterial-based implants hold considerable promise for improving the integration and long-term performance of orthopaedic devices. In addition, they have the potential to significantly reduce the strong risk of complications associated with traditional implants, such as infection and loosening of the implant [13]. These indicate collectively that nano-medicine is all set to play an important role in the advancement of trauma and orthopaedic surgery. Nanostructured materials like NPs, nanofibers, and nano-coatings have allowed solutions more precisely and efficiently, addressing the limitations of conventional therapies in orthopaedic care [1,14].

Bone regeneration and nanostructured scaffolds

Bone regeneration is a complex process that requires the right conditions for cells to grow, specialize, and fit into existing bone structures. Traditional methods, like using bone from the patient (autografts) or from others (allografts), have issues such as causing pain at the donor site, immune system rejection, and limited availability of the bone material [5,15]. Nanostructured scaffolds, which are tiny structures, provide new solutions by closely resembling the natural environment of cells, such as ECM at a very small scale. This helps them better integrate with the body's tissues and improve cell responses [1,5].

Nanohydroxyapatite (nano-HA) is a material that has been widely researched for its role in helping bones heal because it closely resembles the mineral part of bones [16]. It can create a structure that supports tissue healing by providing mechanical support and releasing growth factors. Ming-qi Chen's [1] work has looked into using nano-HA together with polymers like polycaprolactone to make composite structures. This mix has been shown to improve the strength of these structures while still being safe for the body. The research shows that these nano-HA and polymer combinations can have compressive strengths similar to real bones, making them good for use in orthopaedics where bones need to bear weight. Furthermore, this combined approach has been found to encourage the growth and development of bone-forming cells, leading to better bone healing. The study also discusses the use of nano-HA combined with poly(lactic-co-glycolic acid) (PLGA) to create composite scaffolds that provide improved mechanical strength while maintaining biocompatibility [1].

Yi et al.'s research highlights the potential of nanostructured scaffolds in helping to repair large bone injuries that cannot heal on their own. These scaffolds copy the natural layered structure of bone, providing a support system that encourages the growth and development of stem cells from bone marrow [5]. Additionally, by adding growth factors like bone morphogenetic proteins (BMP), these nanostructured scaffolds have exhibited remarkable effectiveness in expediting the healing of extensive bone defects, presenting a viable alternative to conventional grafting approaches [5,17]. Research has found that a fibrin scaffold with recombinant human bone morphogenetic protein-2 (BMP-2) inside heparin/polymer NPs keeps the bioactivity of BMP-2, leading to more osteogenic differentiation in human mesenchymal stem/stromal cells (hMSC). The cells showed about a 500% increase in collagen type I and an 80% increase in bone sialoprotein compared to a fibrin hydrogel with regular BMP-2. Also, when the NPs had six times more osteocalcin (OC), the protein structure became more stable and had a better affinity for calcium and hydroxyapatite, suggesting better osteoblast differentiation. These results show that nano-carriers can boost the osteoconductive ability of BMP-2, making bone regeneration therapies more effective [18].

Garimella et al.'s work focuses on collagen-nano-HA hybrid scaffolds that imitate the organic and inorganic parts of bone. Collagen helps cells grow, and nano-HA gives the necessary minerals for bone development, which helps new and old bones connect better and heal faster in injuries [19]. Nanofiber scaffolds, with their large surface area, improve how cells interact with the scaffold and closely resemble the ECM, encouraging cell movement and growth. Tests in animals have shown that these nanofiber scaffolds could be better than traditional porous scaffolds [20]. Another study found that adding nano-silver to scaffolds gives them antimicrobial properties and boosts blood vessel growth, which is important for large-scale bone repair, making nanostructured scaffolds useful in treating orthopaedic injuries [3]. Savvidou et al. highlight nanostructured scaffolds' clinical applications in nonunion fractures, enabling faster scaffold-bone integration and reduced healing times compared to standard bone grafts, underscoring nanotechnology's potential in complex orthopaedic challenges [14].

Nanostructured scaffolds represent a significant advancement, offering a tailored approach to patient-specific needs. They provide structural support, promote cellular activities, and incorporate bioactive elements, making them vital in treating complex orthopaedic injuries and conditions. A summary of various scaffolds is mentioned in Table 1.

**Table 1:** Summary of nanostructured scaffold types and their mechanisms for bone regeneration. Nano-HA: nanohydroxyapatite; PLGA: poly(lactic-co-glycolic acid); BMP: bone morphogenetic proteins; BMP-2: recombinant human bone morphogenetic protein-2; ECM: extracellular matrix.

Scaffold type	Mechanism of action	Reference
Nano-HA/PLGA	Enhances mechanical strength and osteogenic potential, maintains biocompatibility	[1]
Nano-HA/polycaprolactone	Enhanced mechanical strength, suitable for load-bearing applications, promotes osteoblast growth	[1]
Nano-HA/collagen	Mimics ECM structure for better cell differentiation	[5]
Nanostructured scaffolds with BMP	Accelerates healing of large bone defects, promotes stem cell differentiation	[5,17]
Fibrin scaffold with BMP-2 nano-carriers	Preserves bioactivity of BMP-2, increases osteogenic differentiation, enhances protein stability	[18]

Targeted drug delivery systems

NPs and nanomaterials are very promising for delivering drugs directly to areas where bones need to heal. This method in orthopaedics allows for very accurate treatment of infections and inflammation, while also lowering the risk of side effects in the rest of the body. By directly delivering antibiotics, anti-inflammatory drugs, and growth factors straight to the injured area, NPs can keep high levels of these treatments locally and adverse systemic reactions [21].

One study showed that silver nanoparticles (AgNPs) are very good at stopping infections linked to orthopaedic implants. AgNPs are especially good at preventing biofilm growth, which is a frequent problem that compromises implant integration and leads to infection. The study found that implants covered with AgNPs greatly reduced biofilm growth caused by *Staphylococcus aureus*, a common bacteria that causes infections after surgery [22]. For instance, Alt et al. found that bone cement made of poly(methyl methacrylate) (PMMA) with 1.0% AgNPs (5-50 nm) completely stopped the growth of *Staphylococcus epidermidis*, methicillin-resistant *S. epidermidis* (MRSE), and methicillin-resistant *S. aureus* (MRSA). In comparison, PMMA bone cement with 2% gentamicin sulphate only stopped the growth of *S. epidermidis* [23].

Another research by Ordikhani et al. discovered that implants coated with NPs can decrease bacterial growth and steadily release silver ions, offering long-lasting antibacterial benefits. This method is especially helpful for open fractures and severe injuries, where the risk of infection is high. By lowering the need for extended use of antibiotics, these NPs can help avoid problems such as long hospital stays, antibiotic resistance, and side effects, making them useful for orthopaedic treatments [24].

Selenium NPs show potential in orthopaedics because they can kill germs and help bones heal. They damage bacterial cell walls, making it harder for bacteria to stick to implants, and have reduced infection rates by up to 40% in tests, even against tough strains like MRSA [25]. Besides metal NPs, polymer-based NPs have been created for slow-release drug delivery in orthopaedics. Liposome-based treatments can directly deliver growth factors and anti-inflammatory drugs to bone injury sites, using the liposomes' biocompatibility and drug encapsulation capabilities. Studies show that liposome-delivered anti-inflammatory drugs reduce local inflammation by 30% in animals, better than traditional methods of systemic administration [26]. Savvidou et al.'s work shows how biodegradable polymer NPs can deliver antibiotics directly to treat bone infections. The NPs, loaded with antibiotics, could steadily release the drug over 14 days, maintaining therapeutic levels at the infection site. This targeted approach ensures the gradual release of therapeutics and helps maintain a consistent antimicrobial effect, crucial for treating chronic infections like osteomyelitis [14].

These targeted approaches minimize systemic side effects and enhance patient outcomes in managing infections and inflammation associated with orthopaedic trauma, as summarized in Table 2. As research continues to progress, further refinements are anticipated to elevate the precision and efficacy of these treatments, solidifying their integral role in modern orthopaedic therapy.

**Table 2:** Overview of nanoparticle-based drug delivery systems for orthopaedic applications. NPs: nanoparticles; AgNP: silver nanoparticle; PMMA: poly-methyl-methacrylate; MRSA: methicillin-resistant *Staphylococcus aureus*; MRSE: methicillin-resistant *Staphylococcus epidermidis.*

Drug delivery system	Key benefits	Reference
Polymeric NPs with antibiotics	Sustained antibiotic release over 14 days, crucial for chronic infections like osteomyelitis	[14]
AgNP-coated implants	Sustained silver ion release, effective in reducing biofilms, inhibits MRSA and MRSE	[22]
PMMA bone cement with AgNPs	Complete inhibition of *Staphylococcus epidermidis*, MRSE, and MRSA compared to gentamicin-loaded cement	[23]
Selenium nanoparticles	Disrupts bacterial cell membranes, reduces adhesion, and 40% reduction in infection rates against MRSA	[25]
Liposomal formulations	Direct delivery of anti-inflammatory agents, 30% reduction in local inflammation	[26]

Enhanced implant integration and longevity

Establishing long-term integration between orthopaedic implants and the surrounding bone tissue is a crucial objective in orthopaedic surgery, especially in joint replacement procedures. Nanostructured surface modifications have emerged as a promising approach to enhance the osseointegration of implants, subsequently improving their stability and durability.

Zhang et al.'s research focuses on enhancing the connection and longevity of orthopaedic implants by using bioactive and nanostructured coatings. It points out the problems with implant failure, especially from aseptic loosening, and mentions that many joint replacements need follow-up surgeries [27]. For example, a five-year study by Postler and team found that 36.1% of knee replacements were redone due to infection, and 21.9% were due to aseptic loosening [28]. To tackle these problems, the study looks at using nano-HA coatings, which mimic bone minerals to help bone cells attach and grow, and titania nanotubes that improve cell sticking. The rough surface from these nanostructured coatings helps bone cells multiply, making the implant bond better with the surrounding bone. Additionally, it discusses the role of calcium and magnesium functionalization in stimulating natural apatite formation, and the use of biphasic calcium phosphate to improve osteoconductivity. Antimicrobial coatings, such as silver and nitric oxide, are also emphasized for reducing infection risks, which is crucial for maintaining the longevity of implants [27].

Methods such as severe plastic deformation (SPD) can make metal surfaces very smooth at the nanoscale, which helps them work better with the body and be stronger. This is important for implants that need to last a long time. Adding nanoscale materials, like carbon nanotubes, to a strong plastic called ultra-high molecular weight polyethylene (UHMWPE) makes it more resistant to wear, which could make joint replacements last longer. Special bone cement with tiny particles can slowly release antibiotics, lowering the risk of infections and making implants more successful and long-lasting. These improvements in nanotechnology help implants work better right away and reduce problems, making orthopaedic implants last longer [6].

Orthopaedic oncology and nanomedicine

The area of orthopaedic oncology has made big improvements with the use of nanomedicine, which opens up new ways to treat bone cancers like osteosarcoma and bone metastases. NPs, as discussed earlier in the paper, offer a means to deliver chemotherapeutic agents directly to tumour sites, improving drug concentration at the target while minimizing drug toxicity [24,29].

Orthopaedic implants are very important for fixing bone problems after removing cancerous areas. However, regular implant materials, though good at holding bones together, do not stop cancer from coming back or spreading. New research shows that selenium, a special element, could make orthopaedic implants better at fighting cancer. Selenium coatings, when made very tiny, can tell the difference between bad and good bone cells, stopping the bad ones from growing and helping the good ones work better where the implant meets the bone [30]. Another promising avenue of research involves the development of nanostructured magnesium alloy implants. Recent investigations have revealed that these materials, characterized by enhanced grain refinement, exhibit notable anti-tumour effects (increased bone adhesion, calcium deposition, bone proliferation, and alkaline phosphatase activity). In particular, human osteosarcoma cells exposed to these nanostructured surfaces demonstrated reduced viability and adhesion [31].

PLGA NPs that carry anticancer drugs and target bones provide a focused way to treat and prevent bone metastases. Research has shown that PLGA NPs coated with alendronate are safe for blood, do not cause red blood cell damage or affect blood clotting, and are not harmful to bone marrow cells, blood vessel cells, or bone-forming cells [32,33]. Cenni et al. found that PLGA NPs loaded with doxorubicin and coated with alendronate were just as effective, or even more effective than the free drug in shrinking tumours and preventing bone damage from metastases [34]. Similarly, Pignatello et al. reported a significant decrease in metastases with these NPs compared to free doxorubicin, likely because of alendronate's ability to prevent bone damage [33].

Nanotechnology enhances cancer diagnosis by using nanoparticle-ligand complexes that bind to specific genetic mutations, enabling detailed cellular imaging. This technique can identify a malignancy's metastatic potential early on, for example, by targeting the p15 gene linked to lung metastasis in osteosarcoma. Combining this with nanotechnology-based drug delivery allows earlier chemotherapy initiation, potentially reducing patient morbidity [6,14]. For example, Surender et al. developed europium-emitting, surface-modified gold NPs to serve as MRI contrast agents. These NPs were designed to automatically accumulate on calcium ion-rich surfaces, such as those found in fatigue-induced bone microdamage, enabling targeted labelling of microcracks within bones. This technique allows for precise detection of bone mass and quality, which can help identify areas at risk of fractures, facilitating targeted early intervention [35].

Nanotechnology in sports medicine

Cartilage repair in regenerative medicine has been widely researched because adult cartilage does not heal well on its own. Nanotechnology has improved mesenchymal stem cell (MSC) treatments by using safe materials like hyaluronic acid and nanofibers. These materials show good results for fixing cartilage and helping it grow properly. Clinical tests have shown that using tiny scaffold structures can successfully fill in cartilage and bone defects, but more studies are needed. Also, nanotechnology and drug delivery methods have the potential to reduce scar tissue and make tendons heal better and stronger. Nanocomposite scaffolds are helping to improve tendon tissue engineering [6,25,36].

More studies are being conducted in both cartilage regeneration and tendon healing using nanotechnology to enhance tissue repair and regeneration. Nanomaterial-based scaffolds have shown positive effects on cell adhesion, proliferation, and phenotypic selection of chondrocytes, while controlled release of mitomycin-C, a chemotherapy agent, using hydrosol NPs has helped reduce tendon adhesion formation [37]. For their ability to improve healing, mechanical stability, and viability in tendon tissue engineering, the clinical potential of nanotechnology in promoting tissue regeneration and healing is showcased [6,38]. For example, a study by Huegel and colleagues showed that using a special nanoscale scaffold in shoulder surgery on rats helped the tendons heal better and stay stronger. This shows that nanotechnology has a lot of promise for helping tissues heal and regenerate [39].

Future perspectives and challenges

Nanotechnology has also become important in medicine, where large research funding has increased over time. Although the benefits seem rather obvious, the clinical translation of such findings from the laboratory has remained quite difficult. Advancements in nanomaterials and cellular interaction studies are required. Improved design methods have hence targeted improving nanomaterial integration into implants in ways that guarantee safety. Initial studies have indicated possible benefits, but long-term health considerations call for further investigations. Most such trials hardly get translated into clinical practices due to regulatory approval and the high costs of trials. Besides, very few nanotech research studies have focused on health effects; this puts a vital need for further clinical research on their safety and effectiveness [40-42].

While nanotechnology shows promise, it faces challenges in application in orthopaedics. Major concerns relate to the toxicity of NPs and the indeterminate behaviour of metals at a nano level, since even in metal-on-metal hip arthroplasty, it has raised problems. Preliminary studies showed lung, systemic, and oxidative stress problems. Besides, there are regulatory, manufacturing, and cost-related barriers. These would imply that when balanced legislation, innovative manufacturing, and availability can answer the various challenges, nanotechnology does have a bright future ahead in orthopaedics surgery [40,41,43].

## Conclusions

Nanotechnology has advanced the frontiers of trauma and orthopaedic surgery further into new dimensions, including bone regeneration, infection management, and oncology. Nanostructured scaffolds, precision-targeted drug delivery systems, and bioactive coatings have shown promise so far to improve clinical outcomes through enhanced tissue integration, specific therapeutic delivery, and accelerated healing processes. With these advances notwithstanding, the clinical applications remain bedevilled by the problems of toxicity of nanoparticles, unpredictability of the behaviour of nanoscale materials, and generally strict regulatory environments. The toxicity from nanoparticles generated through wear and the huge expenses of clinical trials remain important obstacles towards wider clinical acceptance. Again, despite all the challenges, nanotechnology has shown great promise towards further advances in orthopaedic treatment, opening up new avenues in treating pathologies like cartilage, tendon, and orthopaedic oncology. Now, what needs to be done is the designing of long-term safety studies, cost-effective production processes, and rational balanced regulatory policies so that full benefits from nanotechnology are exploited. Large continuous investment and rapid innovation in nanotechnology will drive an orthopaedic therapy revolution towards more active, targeted, and safer treatments for a wide range of musculoskeletal conditions.
